# Necrotizing sialometaplasia of the tonsillar pillar. An unusual case

**DOI:** 10.4317/jced.57341

**Published:** 2021-02-01

**Authors:** Julissa-Brillit Hernández-Flores, Edmundo Santos-Jaimes, Luis-Alberto Gaitán-Cepeda

**Affiliations:** 1 Second year resident of Oral pathology program, Graduate and Research Division, Dental School, National Autonomous University of Mexico. Mexico city, Mexico; 2 Professor of Department of Oral Pathology and Oral Medicine, Graduate and Research Division, Dental School, National Autonomous University of Mexico. Mexico city, Mexico; 3 Full time Professor of Department of Oral Pathology and Oral Medicine, Graduate and Research Division, Dental School, National Autonomous University of Mexico. Mexico city, Mexico

## Abstract

Necrotizing Sialometaplasia (NS) is a rare, benign, self-limited, inflammatory and necrotizing reaction of the salivary glandular tissue. Due to the clinical picture (a painful ulcer with well-defined edges), and histopathological characteristics (nuclear and cellular pleomorphism of ductal cells) NS can mimic a malignant lesion. The correct diagnosis is important because NS shows a spontaneous resolution and therefore no further treatment is needed. We report a very unusual case of spontaneous and recurrent NS located on the anterior tonsillar pillar in a 43-year old man, which clinically mimics a malignant lesion. The clinician should be aware to include NS in the differential diagnosis of ulcers in soft palate and tonsillar pillars.

** Key words:**Minor salivary gland, necrotizing sialometaplasia, oral ulcer, squamous cell carcinoma, tonsillar pillar.

## Introduction

Necrotizing sialometaplasia (NS) is a rare (1), benign, self-limited, inflammatory and necrotizing reaction of salivary glandular tissue that represents between 0.03% and 1% of all oral biopsies (2). NS is the result of an ischemic event of the salivary glandular acini vasculature, associated to predisposing factors principally smoking, intake alcohol consumption, wear dental prostheses, recent oral surgery and traumatic injuries (1,3). Although the most common location of NS is hard palate (3), there are cases reported in lower lip, tongue, retromolar region, buccal mucosa, and tonsils (4,5). NS present a slight predisposition for males (male:female ratio 2:1) with an age average of 46 years (2). Clinically, NS is presented as a single and painful ulcer (6) with a necrotic fundus and irregular, elevated and erythematous edges that heals spontaneously in 4 to 12 weeks (7), The recurrence in NS is a very rare event. Histopathological features include squamous metaplasia of ducts and acini, coagulative necrosis with preservation of the lobular architecture, lobar infarction and pseudoepitheliomatous hyperplasia (8).

A very uncommon case of NS due to its location (anterior tonsillar pillar), history of recurrence and not-known predisposing factor, which clinically mimics a malignant lesion, is presented.

## Case Report

A forty-three-year-old male was referred by a private physician to a teaching Oral Medicine Clinic, because he had “a wound on the palate that does not heal”. The medical antecedents of the patient were not relevant for the case. At anamnesis, the patient reported that two months ago, he noticed a small ulcer located in the posterior area of the soft palate painful while swallowing. The ulcer grew in a continuous way, decreasing painful symptoms. The patient referred that 2 years ago, he suffered two similar lesions in the same area of the current ulcer, with an interval of appearance within three months approximately, in both times remitted in approximately two weeks. He denied any kind of traumatism or the use of recreative drugs.

**Figure 1:**
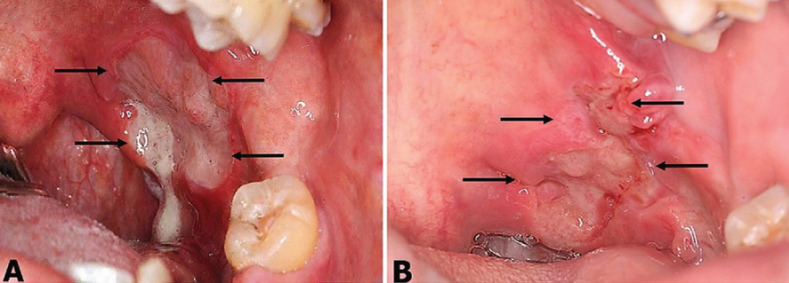
Intraoral view. A) Ulcer at the anterior tonsillar pillar. Notice their irregular shape (black arrows) with edges ill-defined and a white-yellow background (fibrin). B) Clinical appearance 7 days after the incisional biopsy. Observe the notable reduction in ulcer size (black arrows).

At intraoral clinical examination, an ulcer of approximately 3.5 cm in diameter located at the anterior tonsillar pillar was observed. The ulcer was irregular in shape with elevated, erythematous, slightly indurated and poorly defined edges. The bottom of the ulcer was white-yellowish in color with fibrin (Fig. 1A). At palpation, the ulcer was slightly painful. With a presumptive clinical diagnosis of oral squamous cell carcinoma versus necrotizing sialometaplasia, an excisional biopsy was performed. The surgical act was done under local anesthesia without complications. The surgical specimen was fixed in 10% buffer formaldehyde to be sent to the histopathological diagnosis service for processing, observation and diagnosis.

**Figure 2:**
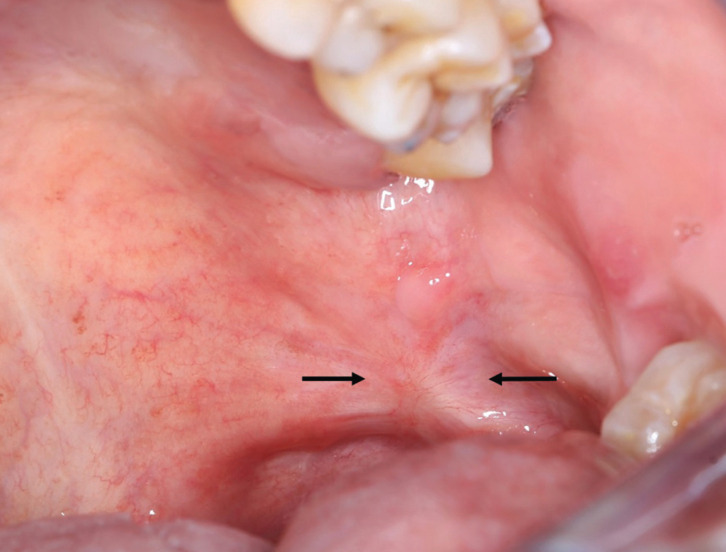
Clinical appearance 37 days after the incisional biopsy. Note the complete healing of the ulcer (black arrows).

Microscopically, the sample was covered by a stratified squamous epithelium, which showed a loss of continuity compatible with an ulcer. Underlaying the epithelium, salivary gland tissue was observed. The sero-mucous salivary acini retained its lobular architecture; however, the presence of coagulative necrosis was identified (Fig. 2A). A severe periductal inflammatory lymphoplasmacytic infiltrate was observed (Fig. 2B) surrounding the salivary excretory ducts. Notably the salivary ducts showed squamous metaplasia (Fig. 2C). A diagnosis of necrotizing metaplasia was emitted.

**Figure 3:**
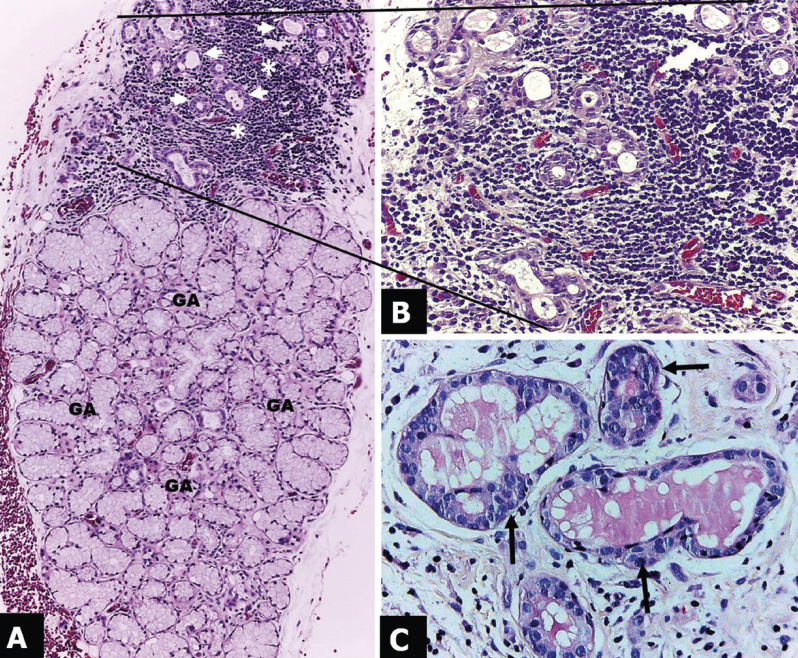
Microscopic aspects. A) Panoramic view of minor salivary gland that shows preserved lobular architecture, glandular acini (GA) with coagulative necrosis, salivary ducts (white arrows); and severe inflammatory infiltrate (white asterisk); hematoxilin & eosin staining. 100X. B) Close up of figure A zone (black lines). Severe inflammatory infiltrate with lymphocyte predominance surrounding the salivary ducts (periductal arranged); hematoxilin & eosin staining. 200X. C) Salivary ducts with squamous metaplasia (black arrows) immersed in an irregular dense connective tissue; hematoxilin & eosin staining. 400X.

At subsequential 7 day-postsurgical follow-up appointment, the patient showed a noticeable process of healing of the surgical area (Fig. 1B). He did not attend his 14th and 28th -day follow-up appointments. At 37th day posterior to surgical event patient attended to his appointment; he was asymptomatic and the area was completely restored (Fig. 3). Currently, the patient is free of oral lesions.

## Discussion

The present report describes an uncommon case of spontaneous and presumably recurrent tonsillar NS. To our knowledge, in the scientific literature only three cases of NS have been reported at tonsils: two cases at tonsillar pilli, and one case at tonsillar fossa (4,5). The present case represents the fourth case of tonsillar NS. All four cases of tonsillar NS are men between 19 – 48 years old. To date, we do not have explanation for this apparent topographic relationship with gender, and casualty should not be ruled out.

NS is generally associated with a predisposing or triggering factor; however, spontaneous lesions have been reported (8,9). In the present case, we did not find clinical evidence or any surgical antecedent that could suggest its probable cause.

It has been reported that NS does not show recurrence (5). To our knowledge the scientific literature shows only 1 cases of recurrent NS located at the nasal cavity (10). In the present case and despite the previous physicians whom attending the patient does not have photographic records of the presumed primary lesion, the actual lesion recurred approximately 6 months later at the same anatomical site, so the possibility of recurrence cannot be ruled out either. Therefore, a long-term follow-up of the patient is required.

Clinically and histopathologically NS can mimic a malignancy, principally oral squamous cell carcinoma and mucoepidermoid carcinoma. Clinically, NS is characterized by a superficial ulcer with or without indurated edges (8). Malignant ulcers due to OSCC or mucoepidermoid carcinoma are deep, frequently involves bone, with indurated borders, however they do not show a rapid growth pattern, which present NS (2,3,11,12). Histologically, NS is characterized by ductal squamous metaplasia, where reactive atypia could be observed resemble muco-epithelial malignant nests observed in mucoepidermoid carcinoma (3,4,7,8). On the other hand, NS is also able to mimic OSCC, due to pseudoepitheliomatous hyperplasia, and mainly, squamous metaplasia in superficial excretory ducts that could resemble infiltrating malignant epithelial islands or epithelium nests in connective tissue (3,8). To differentiate NS from these malignant neoplasms, some key points can be considered in the histological aspect of NS: preservation of the lobar architecture, absence of abnormal mitoses, absence of marked cellular pleomorphism, and absence of vascular or neural invasion (3,8). It has been suggested that immunohistochemical staining technique may be useful for establishing the diagnosis of NS. In such way, anti-p53 and anti-ki-67 immunoreactivity should be absent in NS but present in Carcinomas (4). However, H-E staining remains the gold standard.

Others clinical differential diagnoses included syphilitic gum (13), traumatic ulcerative granuloma with stromal eosinophilia (TUGSE) (14) and major recurrent aphthous stomatitis (11). Syphilitic gummas may also be presented as an ulcer with loco-regional adenopathy, which was not identified in the present case (13). TUGSE shows a slight female predominance and association with trauma, but unlike NS, its presentation usually is in tongue (14). Major recurrent aphthous stomatitis was included in the differential diagnosis due to its clinical characteristics of ulcers of considerable size and recurrence. However, in the present case only one ulcer was present and in the same anatomical site. In the three former diseases even though their clinical aspect could be similar to NS, their histopathological characteristics are distinctives and allows to establish a correct differential diagnosis.

The present case of NS constitutes an unique case due to its location, absence of predisposing factors and its apparent capacity of recurrence. It also shows that a benign lesion (NS) located in an uncommon site constitutes a diagnostic challenge. Because NS can mimic clinically and histopathologically a malignant neoplasm, the clinician must be alert to the presence of a sudden ulcer in the tonsillar region and perform a meticulous examination to avoid misdiagnosis and unnecessary invasive treatments.
